# Camp Roosevelt—Boy Builder

**Published:** 1923-02

**Authors:** F. L. Beals

**Affiliations:** U. S. Army, Chicago, Ill.


### "CAMP ROOSEVELT—BOY BUILDER"

BY MAJOR F. L. BEALS, U. S. ARMY, CHICAGO, ILL.

It is obvious to this enlightened age that without good health success can not be attained, whether it be success in business or social life, or success in the simple enjoyment of living. Therefore, in considering the growing popularity of camp life as the best medium for the proper solution of the summer vacation period, good health must be considered as a pre-requisite to the success of the camping excursion. Educators and thinking men and women the country over are accepting the summer camping idea as the ideal way, especially for boys and girls, to spend the summer months.

At Camp Roosevelt, the national "boy-building" institution which is the first summer camp to be operated and conducted under the auspices of a public institution, a great deal of attention is given to health and sanitation. Boys coming into the camp are given an examination as to their physical fitness and possible breakdown under camp routine. The First Aid Chapter of the American Red Cross of Chicago maintains three physicians and a nurse at the camp at all times, to take care of emergencies and to teach the boy how to apply first aid and emergency measures.

The general health rules, which are fully observed, preclude any serious difficulties, and about the most dangerous troubles brought to the attention of the medical staff are blistered feet, cuts, bruises and other minor afflictions incident to camp life. As far as that goes, a frequent foot inspection is held, to prevent any serious foot trouble or infection.

For the past two years, the Public Service Committee of the Chicago Dental Society was instrumental in sending six dentists to the camp to inspect the teeth of the boys. It is planned to carry on this work every summer. During the summer of 1921, the first summer this program was effective, of the thousand and more boys who had their teeth examined, 509 boys were found with teeth which could be marked "good," 381 "fair," and 98 "poor." The average cavities per company of cadets amounted to three. The past summer, because of the good seed which had been sown the previous summer, the number of cavities was indeed negligible among the boys examined, while the majority of teeth examined came under the mark of "good." This it is felt, is a remarkable increase, due entirely to the good work of the visiting staff during 1921.

Dr. Dan U. Cameron, Chairman of the Public Service Committee, believes that such instruction carried on at camps throughout the country would have a tendency to improve the teeth of the coming generation ninety per cent.

In line with the teaching of mouth hygiene, each boy in camp is given thorough instruction in how to brush his teeth. Toothbrush drill is also part of the program of the camp.

One other point, which has a direct bearing on the health of the camp, is the matter of morale. The healthy camp must have a healthy moral tone, and this is again particularly true with reference to a camp for boys. This part of the work at Camp Roosevelt is entrusted to the officers and instructors, and to the Y. M. C. A. secretaries. The secretaries supply speakers, music, moving pictures, stationery, books, and the like—all helpful forms of service which go far to make camp life more enjoyable and more wholesome.

When it is remembered that boys from all parts of the country assemble here during the summer months, ranging in ages from ten to twenty, it is obvious that an elastic program must be arranged to meet the differing needs of so cosmopolitan a group. To take care of this, a program has been planned, in three sections; the summer schools division, which includes seventh and eighth grade and complete high school subjects, the R. O. T. C. or military division for boys fourteen years and over, and the Junior Camp, for the younger lads. Each boy is placed in the group where he will be best fitted, and where he will come in close contact with other boys of his age and type.

The man who is responsible for the Camp Roosevelt Idea of "building better boys" is Major F. L. Beals, U. S. A., Professor of Military Science and Tactics and Supervisor of Physical Education in the Chicago Public High Schools. Realizing the need for a great playground where boys from all parts of the country could commingle and meet on a common ground, where they could derive a maximum of health and character-building benefits, he founded Camp Roosevelt, and secured the assistance of the U. S. War Department of the Government, the Chicago Board of Education, the American Red Cross, Y. M. C. A., Chicago Dental Society and like organizations to carry on this worthy work. The camp is not operated for profit, but is intended to benefit the average American lad, not a select few. The financial burden is assumed by a committee of representative business men who have formed the Camp Roosevelt Association, and who yearly contribute the sum needed to carry on the enterprise.

Major Beals is always glad to confer with parents regarding their "boy-problems," and inquiries directed to his office at the Board of Education, 460 South State Street, Chicago, will receive immediate response.

Many of our readers undoubtedly have growing sons, and the summer vacation period presents a difficult problem, as to the best means of supervising and training them when freed of the restraining influence of school routine. To such, it is suggested that an earnest study of the Camp Roosevelt Plan for "building better boys" would prove of interest and education. It is the most progressive move of these times for the development of better citizenship.

